# Simulated Data for Genomic Selection and Genome-Wide Association Studies Using a Combination of Coalescent and Gene Drop Methods

**DOI:** 10.1534/g3.111.001297

**Published:** 2012-04-01

**Authors:** John M. Hickey, Gregor Gorjanc

**Affiliations:** * School of Environmental and Rural Science, University of New England, Armidale, 2351 New South Wales, Australia; † Department of Animal Science, Biotechnical Faculty, University of Ljubljana, 1230 Domžale, Slovenia

**Keywords:** genome-wide association studies (GWAS), shared data resources, GenPred, simulation method, quantitative trait loci (QTL), pedigrees

## Abstract

An approach is described for simulating data sequence, genotype, and phenotype data to study genomic selection and genome-wide association studies (GWAS). The simulation method, implemented in a software package called AlphaDrop, can be used to simulate genomic data and phenotypes with flexibility in terms of the historical population structure, recent pedigree structure, distribution of quantitative trait loci effects, and with sequence and single nucleotide polymorphism-phased alleles and genotypes. Ten replicates of a representative scenario used to study genomic selection in livestock were generated and have been made publically available. The simulated data sets were structured to encompass a spectrum of additive quantitative trait loci effect distributions, relationship structures, and single nucleotide polymorphism chip densities.

Simulation studies have made important contributions to the advancement of animal and plant breeding. With many breeding programs now incorporating genomic information at great expense, simulation is both useful and necessary to compare, at low cost, the potential that different analysis methods have to increase the accuracy of estimating breeding values and to compare the alternative structures of breeding programs. Furthermore, simulation can be used to test and benchmark software packages. Recently, many alternative strategies for simulation have been applied within the context of livestock. These strategies use different ways to simulate data, have distributions of quantitative trait loci (QTL) effects, and have different relationship structures. This complicates the comparison of the results and conclusions drawn from the different studies. The first objective of this note was to describe a simple simulation method that can be used to simulate animal or plant genomic data and phenotypes with flexibility in terms of historical population structure, recent pedigree structure, distribution of QTL effects, and with sequence and single nucleotide polymorphism (SNP)-phased alleles and genotypes. The second objective of this note was to provide a set of publically available simulated data sets that cover a spectrum of QTL distributions, relationship structures, and SNP densities. The data were simulated to represent a livestock population and mimic some of the scenarios in which genomic selection is applied.

## Materials and Methods

### Method of simulation

A system to simulate sequence, SNP, and QTL data using a combination of coalescent and gene drop methods was developed. The system is packaged in a Fortran 95 program called AlphaDrop, which calls the Markovian Coalescence Simulator (MaCS) (Chen *et al.* 2009). AlphaDrop has full flexibility in terms of number of chromosomes, QTL, and SNP chips and their density, pedigree structure, and whether the underlying sequence data are outputted. Through the use of MaCS, full flexibility is available in terms of the structure and size of the ancestral population. QTL effects are restricted to being additive and sampled from normal or gamma distributions. MaCS and AlphaDrop are each controlled by a single specification file, examples of which are given in the supporting information, File S1.

Briefly, AlphaDrop starts by setting up the data structures in terms of SNP chips and pedigree. It then calls MaCS, which simulates a sample of haplotypes with sequence information for each chromosome according to the specified ancestral population and mutation and recombination rates. AlphaDrop then drops these haplotypes through a pedigree with a recombination rate assuming 1 recombination event every 100 centimorgans (cM) but no mutation. Internally or externally generated pedigrees can be used. Currently the internal pedigrees are restricted to mammalian species. To simulate data for other species, such as plant species, an externally created pedigree needs to be supplied. The base generation of the pedigree is the most recent generation of the ancestral population simulated using MaCS. Next, the segregating sites are sampled at random to become SNP markers, and a number of SNP chips of different density are provided. The user has full control over the number and density of these chips. The full sequence and phased data can also be outputted if required.

AlphaDrop then selects two samples of segregating sites to possibly become QTL. These are called candidate QTL. The first set comprises a user-specified number of candidate QTL selected at random from across the genome. The second set comprises a user-specified number of candidate QTL selected at random from across the genome with the restriction that the minor allele frequency must be less than a certain threshold. This restriction was designed to facilitate the possibility that QTL have lower minor allele frequency than SNP. Four different traits are then generated assuming an additive genetic model. The first pair of traits is generated using the unrestricted candidate QTL loci. For the first trait (PolyUnres), the allele substitution effect at each QTL locus is sampled from a normal distribution with a mean of zero and standard deviation of one unit. For the second trait (GammaUnres), a random subset of the unrestricted set of candidate QTL loci are selected and the allele substitution effect at each QTL locus is sampled from a gamma distribution with a user-specified shape and scale parameter and a 50% chance of being positive or negative. The second pair of traits (PolyRes and GammaRes) is generated in the same way as the first pair except that the candidate QTL comprise a set with the restriction that their minor allele frequency could not exceed a user specified threshold.

Phenotypes with user-defined heritability are generated for each trait. To ensure that the heritability of the four traits remains constant, the residual variance is scaled relative to the variance of the breeding values of individuals in the base generation of the pedigree, which was given by **a**′**a**/(*n* − 1), where **a** is a vector of breeding value of individuals in the base generation and *n* is the number of individuals in that generation.

AlphaDrop efficiently stores sequence information, and this makes the simulation of sequence data in large pedigrees computationally feasible. Gametes comprise strings of 0s and 1s, representing SNP alleles. Gametes can therefore be thought of as large binary numbers and represented as integers. AlphaDrop breaks gametes into haplotypes of a certain length. Each haplotype can be represented as long integer, and these long integers are only decompressed into their binary numbers where a recombination occurs.

### Simulated data sets

Ten replicates of a livestock data structure were simulated. The structure was designed to cover a spectrum of QTL distributions, relationship structures, and SNP chip densities and to mimic some of the scenarios in which genomic selection is applied. In each replicate sequence data for 4000 base haplotypes for each of 30 chromosomes was simulated using the MaCS (Chen *et al.* 2009). The 30 chromosomes were each 100 cM in length comprising approximately 10^8^ base pairs and were simulated using a per site mutation rate of 2.5*10^−8^ and an effective population size (Ne) of 100 in the final generation of the sequence simulation. The reduction of Ne in the preceding generations was modeled with a Ne 1000 years ago of 1256, a Ne 10,000 years ago of 4350, and a Ne 100,000 years ago of 43,500 with linear changes in between. This reflects estimates by Villa-Angulo *et al.* (2009) for the Holstein population.

A pedigree was simulated comprising 10 generations of individuals, with 50 sires per generation, 10 dams per sire, and 2 offspring per dam. Base individuals in the pedigree had their gametes randomly sampled from the 4000 haplotypes of the sequence simulation allowing for recombination according to the genetic distance using 1% probability of a recombination event per cM. Subsequent generations in the pedigree had their gametes generated through Mendelian inheritance with recombination. The total number of segregating sites across the resulting genome was approximately 1,670,000. A set of 9000 segregating sites were randomly selected from the sequence to be used as candidate QTL loci in two different ways, one a randomly sampled set and the other being a randomly sampled set with the restriction that their minor allele frequency could not exceed 0.30. In addition, a random samples of 60,000 and 300,000 segregating sites was selected from the sequence to be used as SNP on two different SNP chips.

Four different traits were simulated assuming an additive genetic model. The first pair of traits was generated using the 9000 unrestricted candidate QTL loci. For the first trait (PolyUnres), the allele substitution effect at each QTL locus was sampled from a normal distribution with a mean of zero and standard deviation of one unit. For the second trait (GammaUnres), a random subset of 900 of the candidate QTL loci were selected and their allele substitution effects at each QTL locus were sampled from a gamma distribution with a shape of 0.4 and scale of 1.66 (Meuwissen *et al.* 2001) and a 50% chance of being positive or negative. The second pair of traits (PolyRes and GammaRes) was generated in the same way as the first pair except that the candidate QTL loci comprised the 9000 with the restriction that their minor allele frequency could not exceed 0.30. Phenotypes with a heritability of 0.25 were generated for each trait.

### Training and validation data sets

**Figure 1:**
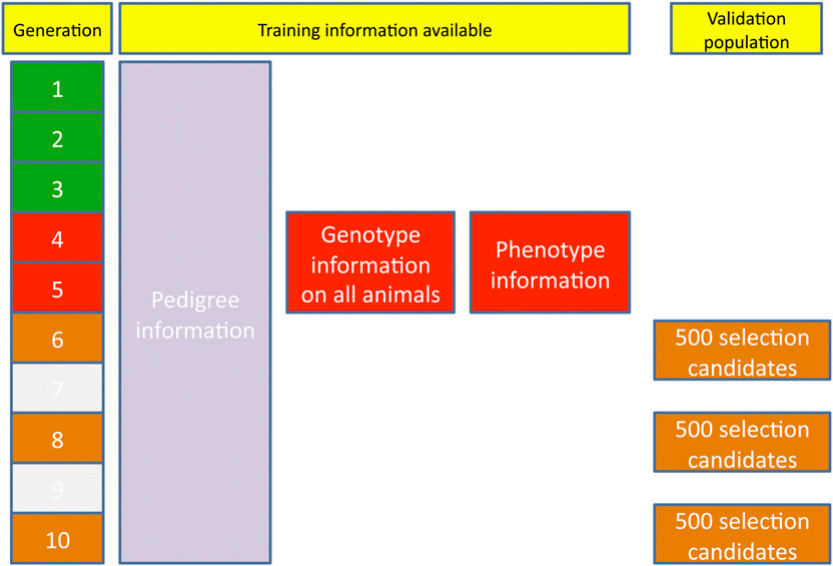
Structure of training and testing data sets.

Subsets of the data were extracted for training and validation. The training set comprised the 2000 individuals in generations 4 and 5 (*i.e.* 1000 animals in each generation). Three validation sets were extracted, consisting of 1500 animals, with 500 animals sampled at random from each of generations 6, 8, and 10. The structure of the training and testing data sets are illustrated in Figure 1.

## Discussion

A system to simulate data for the study of genomic selection in livestock and plants was developed. The system, which combines coalescent and gene drop methods, was designed to be simple and flexible. It makes routine simulation of sequence data for large pedigrees possible. Other genome simulation packages are publically available, such as Fregene (Chadeau-Hyam *et al.* 2008), HaploSim (Coster *et al.* 2010), and QMSim (Sargolzaei and Schenkel 2009). However, given that these packages are based on gene dropping approaches they are less computationally efficient in comparison with the combination of coalescent and gene drop approaches presented here. There are important questions relating to the simulation of genomic data that remain to be resolved. It is not clear whether coalescent or gene drop methods generate realistic genomic data and whether simple additive genetic models are sufficient. Like the simulated data from all other packages, the data simulated by AlphaDrop may not fully reflect the structure of real data. However, the presented approach uses realistic mutation rates, recombination rates, evolution of historical effective population sizes, and numbers of nucleotide base pairs to reflect whole genome level sequence. Simulated data would benefit from having standardized methods to validate its quality. Further development of AlphaDrop is ongoing.

## Supplementary Material

Supporting Information
