# Supplementary material for: Simulated Data for Genomic Selection and Genome-Wide Association Studies Using a Combination of Coalescent and Gene Drop Methods
Source: G3 (Bethesda). 2012 Apr 1;2(4):425–7. doi: 10.1534/g3.111.001297 (PMC3337470; doi:10.1534/g3.111.001297)
Supplement: Supporting Information [file supp_2_4_425__index.html]

Supporting Information 

# Simulated Data for Genomic Selection and Genome-Wide Association Studies Using a Combination of Coalescent and Gene Drop Methods

## Supporting Information for Hickey and Gorjanc, 2012

**Files in this Data Supplement:**

- File S1 - This folder contains ten files

  1) AlphaDrop: executable for Linux

  2) macs: MaCS executable for linux

  3) msformatter: MaCS executable for linux

  4) Seed.txt: a file containing a random seed for initialising AlphaDrop

  5) RunMacs.sh: a shell script called by AlphaDrop when it runs MaCS

  6) AlphaDropSpec.txt: the specification file for AlphaDrop

  7) Pedigree.txt: an example externally supplied pedigree file

  8) MaCsSimulationParameters.xlsx: an excel sheet with which MaCS parameters can be calculated

  9) Ne100.sh: example of what to put into RunMacs.sh (Ne100 population of Hickey et al., 2011 Genetics Selection Evolution)

  10) Ne1000.sh: example of what to put into RunMacs.sh (Ne1000 population of Hickey et al., 2011 Genetics Selection Evolution)

  .zip, 2.1 MB
- Simulated Data - All simulated data available at http://dx.doi.org/10.5061/dryad.nm290
