# Supplementary material for: Simulated Data for Genomic Selection and Genome-Wide Association Studies Using a Combination of Coalescent and Gene Drop Methods
Source: G3 (Bethesda). 2012 Apr 1;2(4):425–7. doi: 10.1534/g3.111.001297 (PMC3337470; doi:10.1534/g3.111.001297)
Supplement: Supporting Information [file supp_2.4.425_FileS1.zip › FileS1/SupplementDataDescription.doc]

The distribution folder for AlphaDrop contains ten files

AlphaDrop – executable for Linux

macs – MaCS executable for linux

msformatter – MaCS executable for linux

Seed.txt – a file containing a random seed for initialising AlphaDrop

RunMacs.sh – a shell script called by AlphaDrop when it runs MaCS

AlphaDropSpec.txt – the specification file for AlphaDrop

Pedigree.txt – an example externally supplied pedigree file

MaCsSimulationParameters.xlsx – an excel sheet with which MaCS parameters can be calculated

Ne100.sh – example of what to put into RunMacs.sh (Ne100 population of Hickey et al., 2011 Genetics Selection Evolution)

Ne1000.sh – example of what to put into RunMacs.sh (Ne1000 population of Hickey et al., 2011 Genetics Selection Evolution)

AlphaDrop is controlled by AlphaDropSpect.txt which is supplied below for the simulated data.

PedigreeStatus	,Internal

NumberGenerations	,10

NumberOfSires	,50

NumberOfDamsPerSire	,10

NumberOfOffspringPerDam	,2

NumberOfChromosomes	,30

ChromosomeLengthMorgans	,1.0

NumberOfSnpChips	,2

nSnpPerChromosme	,2000,10000

WriteFullSequenceOut	,No

TraitHeritability	,0.25

MaxAlleleFreqForQTLForModel3And4	,0.30

NumberOfQTLperChromosomeNormalDist	,300

NumberOfQTLperChromosomeGammaDist	,30

ShapeAndScaleOfGammaDistFor2And4	,0.4,1.66

MaCS is controlled by a single shell script which is supplied below for the simulated data. This creates a population with an effective population size of 100 in the most recent generation. An example for an effective population size of 1000 is also inlcuded in the distribution folder

./ macs 4000 100000000 -t 0.00001 -r 0.000004 -eN 0.03 1.75 -eN 0.06 2.00 -eN 0.13 3.50 -eN 0.25 5.00 -eN 0.50 7.00 -eN 0.75 8.20 -eN 1.00 8.50 -eN 1.25 9.00 -eN 1.50 10.00 -eN 1.75 11.00 -eN 2.00 12.75 -eN 2.25 13.00 -eN 2.50 12.00 -eN 5.00 20.00 -eN 7.50 25.00 -eN 10.00 30.00 -eN 12.50 32.00 -eN 15.00 35.00 -eN 17.50 38.00 -eN 20.00 40.00 -eN 22.50 42.00 -eN 25.00 45.00 -eN 50.00 54.56 -eN 100.00 73.67 -eN 150.00 92.78 -eN 200.00 111.90 -eN 250.00 131.01 -eN 500.00 226.58 -eN 1000.00 417.72 -eN 1500.00 608.86 -eN 2000.00 800.00 2>debug.txt | ./msformatter > haplotypes.txt

tail --lines +6 haplotypes.txt >Intermediate.txt

tail --lines +2 Intermediate.txt > MacsHaplotypes.txt

head --lines 1 Intermediate.txt > TempMap.txt

tail --bytes=+11 TempMap.txt > PhysicalMapInput.txt

grep segsites haplotypes.txt > TempSegSites.txt

tail --bytes=+10 TempSegSites.txt > SegSites.txt

rm Intermediate.txt

rm TempMap.txt

rm TempSegSites.txt

sed -i 's/0/ 0/g' MacsHaplotypes.txt

sed -i 's/1/ 1/g' MacsHaplotypes.txt

cp SegSites.txt FinishedMacs.txt

**Pedigree**

- Name: PedigreeFile.txt
- Columns: 3
- Individual ID (from 1 to number of individuals)
- Father ID (unknown = 0)
- Mother ID (unknown = 0)
- Rows: 10,000
- R code for import:

ped <- read.table(file="PedigreeFile.txt")

colnames(ped) <- c("id", "fid", "mid")

ped$generation <- rep(x=1:10, each=1000)

- Head/tail example:

```
   id  fid  mid
    1    0    0
    2    0    0
  ...  ...  ...
 9999 8173 8188
```

10000 8173 8275

**Phenotype** (training set)

- Name: TrainingPhenotypes.txt
- Columns: 5
- Individual ID (from 3001 to 5000)
- Phenotype value for trait 1 (= PolyUnres)
- Phenotype value for trait 2 (= GammaUnres)
- Phenotype value for trait 3 (= PolyRes)
- Phenotype value for trait 4 (= GammaRes)
- Rows: 2,000
- R code for import:

phe <- read.table(file="TrainingPhenotypes.txt")

colnames(phe) <- c("id", "y1", "y2", "y3", "y4")

- Head/tail example (rounded to one decimal):

```
  id   y1   y2   y3   y4
3001  2.6  0.8 -2.4 -1.2
3002 -0.9  1.7 -0.5 -1.4
 ...  ...  ...  ...  ...
4999 -0.2 -0.8 -0.3 -0.3
```

5000 0.8 0.2 -2.0 -1.4

**Genotype** (training set, SNP chip 1)

- Name: TrainingGenotypesChip1.txt
- Columns: 60,001
- Individual ID (from 3001 to 5000)
- SNP	1 genotype (0, 1, or 2)
- …
- SNP 60,000 genotype (0, 1, or 2)
- Rows: 2,000
- R code for import:

```
genFile <- "TrainingGenotypesChip1.txt"
genT1 <- matrix(data=scan(file=genFile, what=integer()),
                nrow=2000, ncol=60001, byrow=TRUE)
colnames(genT1) <- c("id", paste("SNP", 1:60000, sep=""))
```

- Head/tail example:

```
  id SNP1 SNP2 SNP3 SNP4 SNP5 SNP6 ...
3001    0    1    2    0    2    0 ...
3002    0    0    2    1    2    0 ...
 ...  ...  ...  ...  ...  ...  ... ...
4999    2    0    2    1    2    0 ...
```

5000	0	0	1	1	2	1 ...

**Genotype** (training set, SNP chip 2)

- Name: TrainingGenotypesChip2.txt
- Columns: 300,001
- Individual ID (from 3001 to 5000)
- SNP	1 genotype (0, 1, or 2)
- …
- SNP 300,000 genotype (0, 1, or 2)
- Rows: 2,000
- R code for import:

```
genFile <- "TrainingGenotypesChip2.txt"
genT2 <- matrix(data=scan(file=genFile, what=integer()),
                nrow=2000, ncol=300001, byrow=TRUE)
colnames(genT2) <- c("id", paste("SNP", 1:300000, sep=""))
```

- Head/tail example:

```
  id SNP1 SNP2 SNP3 SNP4 SNP5 SNP6 ...
3001    1    0    1    0    1    2 ...
3002    0    2    1    2    2    0 ...
 ...  ...  ...  ...  ...  ...  ... ...
4999    0    2    2    0    1    2 ...
```

5000	2	1	1	1	0	1 ...

**True breeding values** (validation set)

- Name: ValidationTbvs.txt
- Columns: 5
- Individual ID (a sample between 5000 and 10000)
- True breeding value for trait 1 (= PolyUnres)
- True breeding value for trait 2 (= GammaUnres)
- True breeding value for trait 3 (= PolyRes)
- True breeding value for trait 4 (= GammaRes)
- Rows: 1,500
- R code for import:

tbv <- read.table(file="ValidationTbvs.txt")

colnames(tbv) <- c("id", "a1", "a2", "a3", "a4")

- Head/tail example:

```
  id   a1   a2   a3   a4
5001 -0.2  1.5 -0.3 -1.6
5005 -0.4  1.3 -0.2 -2.1
 ...  ...  ...  ...  ...
9998 -0.8  1.7 -0.7 -2.3
```

9999 -0.3 -0.8 -0.1 -2.3

**Genotype** (validation set, SNP chip 1)

- Name: ValidationGenotypesChip1.txt
- Columns: 60,001
- Individual ID (a sample between 5000 and 10000)
- SNP	1 genotype (0, 1, or 2)
- …
- SNP 60,000 genotype (0, 1, or 2)
- Rows: 1,500
- R code for import:

```
genFile <- "ValidationGenotypesChip1.txt"
genV1 <- matrix(data=scan(file=genFile, what=integer()),
                nrow=1500, ncol=60001, byrow=TRUE)
colnames(genV1) <- c("id", paste("SNP", 1:60000, sep=""))
```

- Head/tail example:

```
  id SNP1 SNP2 SNP3 SNP4 SNP5 SNP6 ...
5001    0    1    2    0    2    0 ...
5005    0    0    2    1    2    0 ...
 ...  ...  ...  ...  ...  ...  ... ...
9998    2    0    2    1    2    0 ...
```

9999	0	0	1	1	2	1 ...

**Genotype** (validation set, SNP chip 2)

- Name: ValidationGenotypesChip2.txt
- Columns: 300,001
- Individual ID (a sample between 5000 and 10000)
- SNP	1 genotype (0, 1, or 2)
- …
- SNP 300,000 genotype (0, 1, or 2)
- Rows: 1,500
- R code for import:

```
genFile <- "ValidationGenotypesChip2.txt"
genV2 <- matrix(data=scan(file=genFile, what=integer()),
                nrow=1500, ncol=300001, byrow=TRUE)
colnames(genV2) <- c("id", paste("SNP", 1:300000, sep=""))
```

- Head/tail example:

```
  id SNP1 SNP2 SNP3 SNP4 SNP5 SNP6 ...
5001    1    0    1    0    1    2 ...
5005    0    2    1    2    2    0 ...
 ...  ...  ...  ...  ...  ...  ... ...
9998    0    2    2    0    1    2 ...
9999    2    1    1    1    0    1 ...
```
